# Activation of amygdala opioid receptors by electroacupuncture of Feng-Chi (GB20) acupoints exacerbates focal epilepsy

**DOI:** 10.1186/1472-6882-13-290

**Published:** 2013-10-29

**Authors:** Pei-Lu Yi, Chin-Yu Lu, Chiung-Hsiang Cheng, Yi-Fong Tsai, Chung-Tien Lin, Fang-Chia Chang

**Affiliations:** 1 Department of Veterinary Medicine, School of Veterinary Medicine, National Taiwan University, No. 1, Sec. 4., Roosevelt Road, Taipei 106, Taiwan; 2 Department of Sports, Health & Leisure, College of Sports Knowledge, Aletheia University, Tainan Campus, Tainan Taiwan; 3 Graduate Institute of Brain & Mind Sciences, College of Medicine, National Taiwan University, Taipei Taiwan; 4 Graduate Institute of Acupuncture Science, College of Chinese Medicine, China Medical University, Taichung Taiwan

**Keywords:** Electroacupuncture, Feng-Chi (GB20), Epilepsy, Amygdala, Opioid receptors

## Abstract

**Background:**

The effect of seizure suppression by acupuncture of Feng-Chi (GB20) acupoints has been documented in the ancient Chinese literature, Lingshu Jing (Classic of the Miraculous Pivot), however, there is a lack of scientific evidence to prove it. This current study was designed to elucidate the effect of electroacupuncture (EA) stimulation of bilateral Feng-Chi (GB20) acupoints on the epileptic activity by employing an animal model of focal epilepsy.

**Methods:**

Administration of pilocarpine into the left central nucleus of amygdala (CeA) induced the focal epilepsy in rats. Rats received a 30-min 100 Hz EA stimulation of bilateral Feng-Chi acupoints per day, beginning at 30 minutes before the dark period and performing in three consecutive days. The broad-spectrum opioid receptor antagonist (naloxone), μ-receptor antagonist (naloxonazine), δ-receptor antagonist (naltrindole) and κ-receptor antagonist (*nor*-binaltorphimine) were administered directly into the CeA to elucidate the involvement of CeA opioid receptors in the EA effect.

**Results:**

High-frequency (100 Hz) EA stimulation of bilateral Feng-Chi acupoints did not suppress the pilocarpine-induced epileptiform electroencephalograms (EEGs), whereas it further increased the duration of epileptiform EEGs. We also observed that epilepsy occurred while 100 Hz EA stimulation of Feng-Chi acupoints was delivered into naïve rats. EA-induced augmentation of epileptic activity was blocked by microinjection of naloxone, μ- (naloxonazine), κ- (*nor*-binaltorphimine) or δ-receptor antagonists (natrindole) into the CeA, suggesting that activation of opioid receptors in the CeA mediates EA-exacerbated epilepsy.

**Conclusions:**

The present study suggests that high-frequency (100 Hz) EA stimulation of bilateral Feng-Chi acupoints has no effect to protect against pilocarpine-induced focal epilepsy; in contrast, EA further exacerbated focal epilepsy induced by pilocarpine. Opioid receptors in the CeA mediated EA-induced exacerbation of focal epilepsy.

## Background

Acupuncture has been used as an alternative medicine to effectively treat patients with chronic pain [1] (e.g., migraines, tension-type headache and peripheral joint osteoarthritis [2]) by manipulating thin needles that have been inserted into the acupoints. Electroacupuncture (EA) is a modified form of acupuncture, which consists of passing an electrical current through needles into the acupoints and controls the acupuncture dose by modifying the frequency and intensity of stimulating currents. Acupuncture had been documented in some Chinese literatures indicating the therapeutic effect in epilepsy, insomnia, and other neurological diseases, in addition to its indication in pain relief. For example, the effect of epileptic suppression by acupuncture of Feng-Chi (GB20) acupoints has been documented in the Lingshu Jing (Classic of the Miraculous Pivot). However, there is a lack of scientific evidence to elucidate its underlying mechanism and to prove the clinical action. Epilepsy is one of the most common and devastating neurological disorders. About seventy percent of patients with epilepsy can be well-controlled with currently available anti-epileptic drugs (AEDs), but seizures still persist in 30% of epilepsy patients who do not respond to any of two to three first-line AEDs despite administration of the carefully optimized drug treatment [3]. Alternative therapies, such as vagus nerve stimulation [4,5], deep brain stimulation (DBS) [6] and acupuncture [7,8], have been considered for treating refractory epilepsy. Several reports demonstrate that acupuncture may suppress seizure activity through the activation of vagus nerve, which subsequently activates the nucleus of tractus solitarius (NTS) [7,8]. Our previous results demonstrate that EA stimulation of bilateral Anmian (EX17) acupoints enhances sleep through the activation of opioid receptors in the NTS [9,10]. Feng-Chi acupoint is anatomically close to the Anmain acupoint. It has been shown that amygdala receives the afferent projection from the NTS [11,12]. Altering the NTS activity changes dynorphin gene expression in the amygdala [13]. Therefore, stimulation of Feng-Chi acupoints may activate vagus nerve and subsequently modify the opioid receptors in the amygdala to achieve its effect in suppressing focal epilepsy. This study was designed to elucidate the effect of high-frequency (100 Hz) EA stimulation of Feng-Chi acupoints in an animal model of focal epilepsy by administering pilocarpine into the left central nucleus of amygdala (CeA).

Discovery of endogenous opioid peptides, including β-endorphin, dynorphin, enkephalin and endomorphin, in the central nervous system (CNS) reveals the mysterious actions of acupuncture, especially in its analgesic effect. It had first been demonstrated that the acupuncture-induced analgesic effect could be blocked by a broad-spectrum opioid receptor antagonist naloxone in both humans and mice [14,15], implicating the role of endogenous opioid peptides. Chang and Pomeranz had revealed that relatively low doses of naloxone only block the analgesic effect induced by low-frequency (4 Hz) of electroacupuncture (EA) stimulation, but not the consequence induced by high-frequency (200 Hz) of EA [16], suggesting that the low-frequency, rather than the high-frequency, of EA increases the release of endogenous opioids. Nevertheless, Han and his colleagues have further shown that the increase of endogenous opioids mediates the analgesic effects induced by both the low-frequency and high-frequency EA stimuli by employing distinct opioid receptor subtype-specific antagonists [17,18]. While μ- and δ-opioid receptors in the spinal cord are dominant in the low-frequency EA-induced analgesia, κ-opioid receptors contribute to the high-frequency EA effects [17,18]. Radioimmunoassay of spinal perfusates from rats receiving various frequencies of EA stimulations further indicates that 2 Hz EA enhances enkephalin (a mixed μ- and δ-opioid receptor agonist) immunoreactivity (IR), but not the dynorphin (κ- opioid receptor agonist) IR. In contrast, 100 Hz EA increases dynorphin IR rather than enkephalin IR [19]. However, whether EA of bilateral Feng-Chi acupoints alters the activity of opioid receptors in the CeA and subsequently changes the epileptic activity in the model of focal epilepsy is not determined. Our current study further elucidated the involvement of CeA opioid receptors in the alteration of epileptic activity induced by high-frequency (100 Hz) EA stimulation of bilateral Feng-Chi acupoints.

## Methods

### Pharmacological agents

Stock solutions of a broad-spectrum opioid antagonist (naloxone hydrochloride (Tocris, Bristol, UK)), a μ-receptor antagonist (naloxonazine dihydrochloride (Tocris)), a δ-receptor antagonist (naltrindole hydrochloride (Tocris)) and a κ-receptor antagonist (*nor*-binaltorphimine dihydrochloride (Tocris)) were dissolved in pyrogen-free saline (PFS). Pilocarpine (1 mg/1 μl) was also dissolved in PFS. The stock solutions were stored at 4°C until use. Our previous results and others have indicated that the appropriate microinjection dosage for naloxonazine, naltrindole and *nor*-binaltorphimine to selectively block μ-, δ- and κ-opioid receptors, without interaction with other opioid receptor subtypes, is within 20 μg [9,10,20,21]. In current study, naloxone, naloxonazine, naltrindole and *nor*-binaltorphimine were microinjected at the dose of 10 μg/μl, which efficiently exhibits their effect of pharmacological blockade according to our previous studies [9,10]. The total volume used for each microinjection was 1 μl.

### Animals

Male Sprague-Dawley rats (250 - 300 g; National Laboratory Animal Breeding and Research Center, Taiwan) were used in this study. Rats were anesthetized by intraperitoneal injection with 50 mg/kg Zoletil® (Virbac, Carros, France). Most of rats (groups 2-8, see the experimental protocol) were surgically implanted with three electroencephalogram (EEG) screw electrodes as previously described [22] and a microinjection guide cannulae directed into the left CeA (AP, 2.8 mm from bregma; ML, 4.2 mm; DV, 7.8 mm relative to bregma). The coordinates were adopted from the Paxinos and Watson rat atlas [23]. Two screw EEG electrodes were placed over the left frontal and parietal lobes of cortices, and a third EEG electrode was placed over the right cerebellum and served to ground the animal to reduce signal artifacts. Rats in the group 1 were implanted with 6 EEG electrodes; three electrodes were implanted in the left frontal, parietal and occipital lobes and the other three electrodes were implanted in the coordinate right hemisphere. One additional reference electrode was placed over the right cerebellum. Insulated leads from EEG electrodes were routed to a Teflon pedestal (Plastics One, Roanoke, VA, USA). The Teflon pedestal was then cemented to the skull with dental acrylic (Tempron, GC Co., Tokyo, Japan). The incision was treated topically with polysporin (polymixin B sulfate – bacitracin zinc) and the animals were allowed to recover for seven days prior to the initiation of experiments. The rats were housed separately in individual recording cages in the isolated room, in which the temperature was maintained at 23 ± 1°C and the light:dark (L:D) rhythm was controlled in a 12:12 h cycle (40 Watt × 4 tubes illumination). Food (5001 rodent diet, LabDiet) and water were available *ad libitum*. All procedures performed in this study were approved by the National Taiwan University Animal Care and Use Committee.

### Experimental protocol

**Figure 1:**
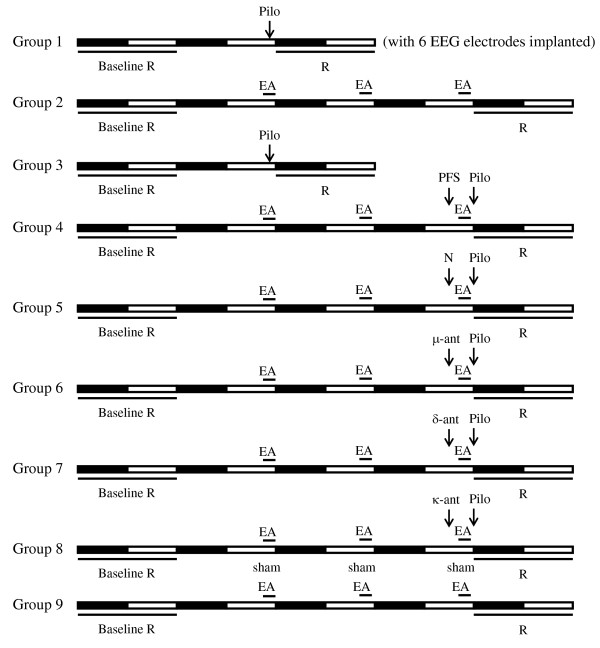
**Schematic representation of the experimental protocol.** Closed bars indicate the dark period and open bars represent the light period of the 12:12 h light:dark cycle. Arrows depict the time of microinjections. R: EEG recording; Pilo: pilocarpine; EA: electroacupuncture; N: naloxone; μ-ant: μ-antagonist (naloxonazine); δ-ant: δ-antagonist (naltrindole); κ-ant: κ-antagonist (*nor*-binaltorphimine).

On the 2^nd^ postsurgical day, the rats were connected to the recording apparatus (see below) via a flexible tether. As such, the rats were allowed relatively unrestricted movement within their own cages. One week after rats had adapted to the 12:12-hour L:D cycle after surgery, a 24-hour undisturbed baseline EEG recordings were obtained beginning at dark onset on the 1^st^ recording day in rats from all groups. Nine groups of rats were used in the study as follows. Group 1 (n = 3) was used to determine the focal epilepsy induced by administration of pilocarpine into the left CeA. Rats in the group 2 (n = 6) received a 30-min 100 Hz EA stimulation of bilateral Feng-Chi acupoints per day, beginning at 30 minutes before the dark period and performing in three consecutive days (the EA group). EEGs were recorded right after the end of the last period of EA stimuli and lasted for 24 hours. Rats in the group 3 (n = 6) were administered with pilocarpine into the left CeA and EEGs were recorded beginning from the dark onset of the L:D cycle (the pilocarpine group). In the group 4 (n = 6), rats received the same EA stimulation protocol as those rats in the group 2 and were respectively administered with PFS and pilocarpine into the CeA before and after the last period of EA stimulation (the PFS + EA + pilocarpine group). Rats in the group 5 (n = 6) were used to determine the effects of opioid receptor antagonist, naloxone, on the 100 Hz EA-induced alterations in the epileptiform EEGs (the naloxone + EA + pilocarpine group). Rats in the group 6 (n = 6), 7 (n = 6), and 8 (n = 6) were respectively used to depict the effects of μ-receptor antagonist (naloxonazine, the naloxonazine + EA + pilocarpine group), δ-receptor antagonist (naltrindole, the natrindole + EA + pilocarpine group) and κ-receptor antagonist (*nor*-binaltorphimine, the *nor*-binaltorphimine + EA + pilocarpine group) on the 100 Hz EA-induced alterations of the epileptiform EEGs. Rats in groups 5-8 received the similar protocol as those in the group 4, except that naloxone (10 μg/μl), naloxonazine (10 μg/μl), naltrindole (10 μg/μl) and *nor*-binaltorphimine (10 μg/μl) were administered into the CeA before the last period of EA stimulation in the group 5, 6, 7 and 8, respectively. Rats in the group 9 (n = 6) had the similar protocol as those in the group 4, except that rats received the sham EA stimulation (the sham EA group). The schematic representation of the experimental protocol was depicted in Figure 1. When 100 Hz EA was given (see later), all rats were lightly anesthetized with 29/4 mg/kg of ketamine/xylazine (one third of the dose which we used for surgery in our previous studies [9,10,22]), after which rat woke up in 30 to 35 minutes. A 30-min period of EA stimulation was administered before the onset of the dark period per day and was applied in three consecutive days. The anesthetization was given 30 minutes prior to the dark period onset and lasted for 30 minutes. The 100 Hz EA stimulus was delivered via the bilateral insertion of stainless needles (32 gauge × 1”, Shanghai Yanglong Medical Articles Co.) on Feng-Chi (GB20) acupoints in the depth of 2 mm. The stimulus consisted of a train of biphasic pulses (150 μs duration each) of 100 Hz with intensity of 1 mA, and was delivered by Functions Electrical Stimulator (Trio 300, I.T.O., Japan). The location of Feng-Chi acupoints in the rat is anatomically similar to that in human. The acupoint of Feng-Chi (GB 20) locates in the depression between the upper portion of m. sternocleidomastoideus and m. trapezius in human. Sham EA was performed by stimulation of a non-acupoint located at the ventral conjunction between the forelimb and the trunk as previous described [24].

### Apparatus and recording

Signals from the EEG electrodes were fed into an amplifier (Colbourn Instruments, Lehigh Valley, PA; model V75-01). The EEG was amplified (factor of 5,000) and analog bandpass was filtered between 0.1 and 40 Hz (frequency response: ±3 dB; filter frequency roll off: 12 dB/octave). These conditioned EEG signals were subjected to analog-to-digital conversion with 16-bit precision at a sampling rate of 128 Hz (NI PCI-6033E; National Instruments, Austin, TX). The digitized EEG waveforms were stored as binary computer files pending subsequent analyses. Postacquisition determinations of the onset and the duration of the EEG seizure occurrence were done by the visual scoring using AxoScope 10 Software (Molecular Devices, Sunnyvale, CA, USA). We defined EEG documented seizures as the visualization of epileptiform spikes with amplitudes of greater than 2 mV appearing in discharges lasting for at least 30 seconds [6].

### Statistical analyses for experiment protocol

All values acquired from the EEG recordings were presented as the mean ± SEM for the indicated sample sizes. Unpaired student t-test for the duration of epileptiform EEGs were performed to analyze and compare the difference between groups. An α level of p < 0.05 was taken as indicating a statistically significant difference.

## Results

### Administration of pilocarpine into the left CeA induces focal epilepsy

**Figure 2:**
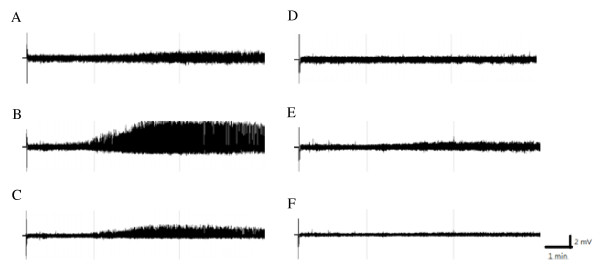
**The focal epilepsy induced by microinjection of pilocarpine into the CeA.** Panels **A**, **B**, **C**, **D**, **E** and **F** represent the EEG signals acquired from the electrodes of left frontal, left parietal, left occipital, right frontal, right parietal and right occipital cortices, respectively. The epileptiform EEGs were predominantly recorded from the electrode of left parietal cortex.

**Figure 3:**
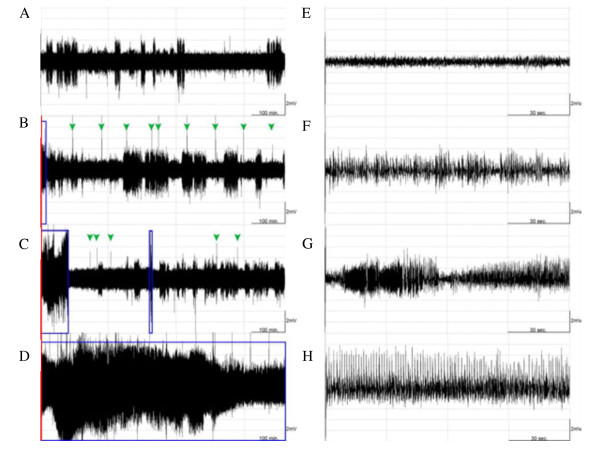
**The effect of 100 Hz EA stimulation of bilateral Feng-Chi acupoints on the epileptic activities.** Panels **A**, **B**, **C** and **D** respectively depict the EEG signals recorded from the naïve rats, the EA group, the pilocarpine group, and the PFS + EA + pilocarpine group, beginning from the dark onset of the dark period. Red lines indicate the time for pilocarpine administration (at the end of the 30-min EA stimulation). The blue boxes represent the epileptiform EEGs. Green arrowheads were the artifacts. The larger amplitudes, with EEG signals less than 2 mV, appeared in panels A, B and C were delta waves, which represent the state of slow wave sleep. Panels **E**, **F**, **G** and **H** were the enlarged figures which were respectively expanded the time scale from the panels A, B, C and D, and were recorded from the beginning of the dark period.

**Figure 4:**
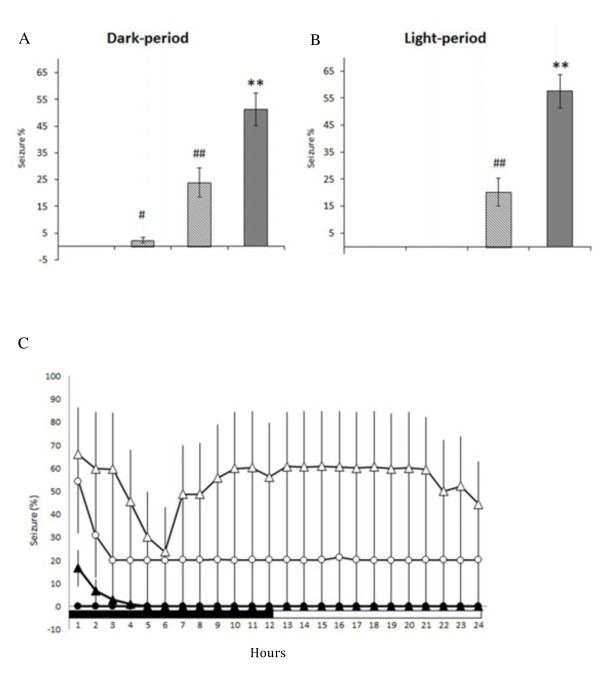
**The summary of 100 Hz EA stimulation of bilateral Feng-Chi acupoints on the epileptic activities. Panel A** depicts the results obtained from the dark period and **panel B** demonstrates the data acquired from the light period. The bars from the left to the right in both panels A and B represent the results obtained from the naïve rats, the EA group, the pilocarpine group, and the PFS + EA + pilocarpine group. In **panel C**, the black circles represent the values obtained from the naïve group, the black triangles depict the results of the EA group, the white circles demonstrate the values obtained from the pilocarpine group, and the white triangles indicate the data acquired from the PFS + EA + pilocarpine group. #: p < 0.05 vs. the naïve rats; ##: p < 0.01 vs. the naïve rats; **: p < 0.01 vs. the pilocarpine group.

The predominant epileptiform EEGs were recorded from the left parietal electrode and some epileptic activities were also acquired from the left occipital electrode when administration of pilocarpine (1 mg) into the CeA in rats of group 1, whereas no epileptic activity was recorded from the rest of four electrodes (Figure 2). The epileptic EEGs were primarily recorded immediately after pilocarpine injection (Figure 2B & Figure 3C), and the epileptic recurrence occasionally happened during a day (Figure 3C). The average of time exhibiting epileptiform EEGs in the pilocarpine group (the group 3) was 23.9 ± 5.4% during the dark period and 20.2 ± 5.2% during the following light period (Figure 4A & 4B). This observation indicates that administration of pilocarpine into the left CeA successfully induces focal epilepsy in rats.

### The effect of 100 Hz EA on pilocarpine-induced focal epilepsy

We determined the effect of 100 Hz EA of bilateral Feng-Chi acupoints on the epileptiform EEG activities induced by pilocarpine. There was no epileptic activity been recorded in the naïve rats without any manipulation (Figures 3A, 3E & 4). We surprisingly found that 100 Hz EA of bilateral Feng-Chi acupoints induced epileptiform EEGs in four out of six (4/6) naïve rats (Figure 3B & 3F). The average of time exhibiting epileptiform EEGs during the 12 hours of the dark period in the EA group (the group 2) was 2.3 ± 1.0%, whereas no epileptiform activity was observed in the following light period (Figure 4A & 4B). The predominant epileptiform EEGs induced by 100 Hz EA were observed during the first two hours after the EA stimulation (Figure 4C). Furthermore, the high-frequency EA stimulation aggravated the amplitude and duration of the epileptic EEG activity induced by pilocarpine (Figures 3D, 3H & 4). The average of time presenting epileptic activities in the PFS + EA + pilocarpine group (the group 4) was significantly enhanced to 51.2 ± 6.1% during the dark period (p < 0.01, when compared to the values obtained from the pilocarpine group) and increased to 57.5 ± 6.1% during the following light period (p < 0.01, when compared to the results acquired from the pilocarpine group; Figure 4A, 4B & 4C). Our previous results have shown that sham EA stimulation did not alter baseline EEGs [9,10]. We also observed that sham EA stimulation did not exhibit effect on pilocarpine-induced epileptiform EEGs (data not shown).

### CeA opioid receptors mediate EA-induced augmentation of focal epilepsy

**Figure 5:**
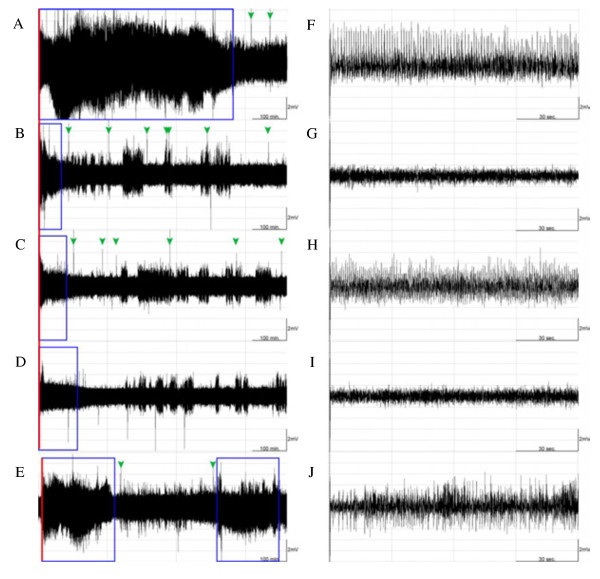
**The effects of naloxone, naloxonazine, naltrindole and *nor*-binaltorphimine on the 100 Hz EA-induced exacerbation of epileptic activities.** Panels **A**, **B**, **C**, **D** and **E** respectively depict the EEG signals recorded from the PFS + EA + pilocarpine group, naloxone + EA + pilocarpine group, the naloxonazine + EA + pilocarpine group, the naltrindole + EA + pilocarpine group, and the *nor*-binaltorphimine + EA + pilocarpine group, beginning from the dark onset of the dark period. Red lines indicate the time for pilocarpine administration (at the end of the 30-min EA stimulation). The blue boxes represent the epileptiform EEGs. Green arrowheads were the artifacts. The larger amplitudes, with EEG signals less than 2 mV, appeared in panels **B**, **C** and **D** were delta waves, which represent the state of slow wave sleep. Panels **F**, **G**, **H**, **I** and **J** were the enlarged figures which were respectively expanded the time scale from the panels **A**, **B**, **C**, **D** and **E**, and were recorded from the beginning of the dark period.

**Figure 6:**
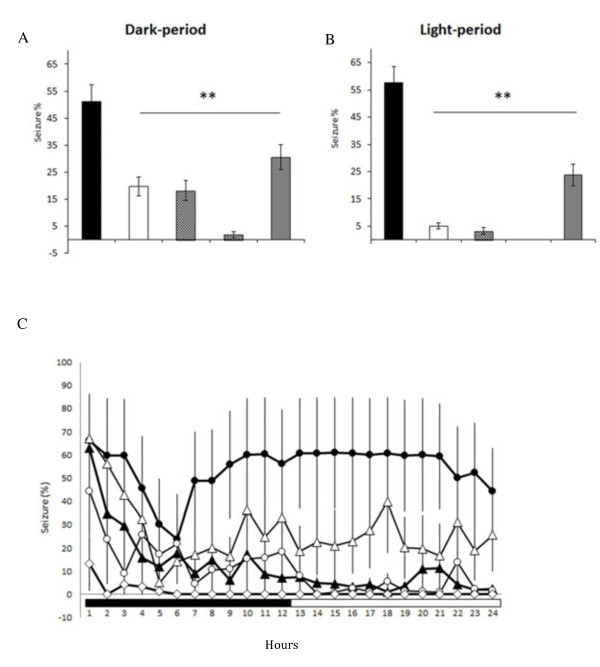
**The summary for the effects of naloxone, naloxonazine, naltrindole and *nor*-binaltorphimine on the 100 Hz EA-induced exacerbation of epileptic activities. Panel A** depicts the results obtained from the dark period and **panel B** demonstrates the data acquired from the light period. The bars from the left to the right in both panels A and B represent the results obtained from the PFS + EA + pilocarpine group, naloxone + EA + pilocarpine group, naloxonazine + EA + pilocarpine group, naltrindole + EA + pilocarpine group and *nor*-binaltorphimine + EA + pilocarpine group. In **panel C**, the black circles represent the values obtained from the PFS + EA + pilocarpine group, the black triangles depict the results of the naloxone + EA + pilocarpine group, the white circles demonstrate the values obtained from the naloxonazine + EA + pilocarpine group, the white diamonds elucidate the results of the naltrindole + EA + pilocarpine group, and the white triangles indicate the data acquired from the *nor*-binaltorphimine + EA + pilocarpine group. **: p < 0.01 vs. the PFS + EA + pilocarpine group.

Application of naloxone (10 μg) significantly reduced the amplitude and duration of EA-induced augmentation of epileptic EEGs (Figure 5B & 5G). The average of time exhibiting epileptic EEGs in the naloxone + EA + pilocarpine group was significantly reduced to 19.7 ± 3.5% during the dark period (p < 0.01, when compared to the values obtained from the PFS + EA + pilocarpine group; Figure 6A) and decreased to 5.0 ± 1.1% during the following light period (p < 0.01, when compared to the data acquired from the PFS + EA + pilocarpine group; Figure 6B). Both naloxonazine (10 μg) and naltrindole (10 μg) exhibited similar effect in reducing the amplitude and duration of EA-induced augmentation of epileptic EEGs (Figures 5 & 6). The average of time presenting epileptic EEGs in the naloxonazine + EA + pilocarpine group and in the naltrindole + EA + pilocarpine group was respectively decreased to 18.2 ± 3.8% (p < 0.01) and 1.9 ± 1.1% (p < 0.01, when compared to the values obtained from the PFS + EA + pilocarpine group) during the dark period, and it was decreased to 3.3 ± 1.3% (p < 0.01) and 0% (p < 0.01, when compared to the data acquired from the PFS + EA + pilocarpine group) during the following light period (Figure 6). *Nor*-binaltorphimine (10 μg) reduced the duration of EA-induced augmentation of epileptic activity, but it exhibited less effect in suppressing the amplitude of epileptiform EEGs (Figure 5E & 5J). The average of time exhibiting epileptic EEGs in the *nor*-binaltorphimine + EA + pilocarpine group was significantly reduced to 30.5 ± 4.7% during the dark period (p < 0.01, when compared to the values obtained from the PFS + EA + pilocarpine group) and decreased to 23.7 ± 4.0% during the following light period (p < 0.01, when compare to the results acquired from the PFS + EA + pilocarpine group; Figure 6).

## Discussion

The goal of this study is to elucidate the effect of EA stimulation of Feng-Chi acupoints on epileptic suppression. Epilepsy can be divided into focal and generalized epilepsy according to classification proposed by International League Against Epilepsy (ILAE). Focal epilepsy is usually subtle and the epileptiform activity starts in one area of brain and may spread to other brain regions. In contrast, generalized epilepsy, which is more severe than focal epilepsy, is result of abnormal brain activity in both hemispheres. In this study, we would like to first determine whether EA of Feng-Chi acupoints suppresses focal epilepsy. Systemic administration of pilocarpine in rats leads to a pattern of generalized seizure and status epilepticus [25]. However, the reliability of focal epilepsy induced by administration of pilocarpine into CeA has been confirmed in this study (Figure 2). We found that epileptiform EEGs were primarily recorded from the left parietal electrode near left CeA, but were not acquired from electrodes implanted on the right hemisphere, when EEG signals were acquired by multiple electrodes on both hemispheres.

Acupuncture and EA has been recommended as an alternative medicine for several therapeutic indications by the World Health Organization (WHO), such as alleviation of pain, reduction of inflammation and management of insomnia. The clinical therapeutic effects and the underlying mechanisms of EA in pain relief has been well elucidated; however, its effects in other aspects, such as neurodegenerative diseases, insomnia and epilepsy, has been less investigated. Feng-Chi acupoint (GB 20), located in the depression between the upper portion of m. sternocleidomastoideus and m. trapezius in human, has been documented in the Lingshu Jing (the Classic of the Miraculous Pivot) and indicated the therapeutic effects in headache, dizziness, hypertension and epilepsy. Acupuncture may become the alternative choice to treat patients with refractory epilepsy who do not respond to the current AEDs. However, the effect of acupuncture in treating epilepsy is controversial. Activation of vagus nerve by EA has been reported as a promising neuroprotective therapy for patients with refractory epilepsy [7,8]. However, bilateral acupuncture of Taichon (LR3), Hegu (LI4) and Baihui (GV20) acupoints did not significantly alter the frequency of seizure occurrence in patients with refractory epilepsy, indicating that acupuncture is not beneficial in patients with refractory epilepsy [26]. A Cochrane review also concludes that no enough scientific evidence supports the effectiveness of acupuncture in epilepsy therapy [27]. To clarify the dispute of acupuncture effect in the epilepsy therapy, we designed this current study to determine the effect of high frequency (100 Hz) EA of bilateral Feng-Chi acupoints in the focal epilepsy induced by administration of pilocarpine into the left CeA. Our results indicated that administration of pilocarpine into the left CeA induced focal epilepsy, however, the pilocarpine-induced epileptiform EEGs were augmented when rats previously received the 100 Hz EA stimuli of Feng-Chi acupoints. This result indicated that high-frequency (100 Hz) EA stimulation of bilateral Feng-Chi acupoints exacerbated pilocarpine-induced epileptic activity, rather than protecting against epilepsy. In fact, our data demonstrated that 100 Hz EA stimulation of bilateral Feng-Chi acupoints induced epileptic activities in the naïve rats, which did not receive any manipulation. The characteristic of epileptogenesis induced by the high-frequency EA stimulation of bilateral Feng-Chi acupoints *per se* may cause the aggravation of pilocarpine-induced epileptiform EEG activities. These observations, since they subvert the functions of Feng-Chi acupoints documented in the Lingshu Jing, surprise us. The possible reasons of contradiction between our findings and the documentation in Lingshu Jing are as follows. First, with or without delivering electrical currents into Feng-Chi acupoints is a fact. The effect of epileptic suppression documented in Lingshu Jing is manipulated by dry needling, whereas the exacerbation of epilepsy we observed in this study was the results after EA with delivering currents into acupoints. Second, different stimulation frequencies may differ the outcomes. It is worthy to investigate the effect of different EA stimulation frequencies, especially for the lower frequency (e.g., 10 Hz), on the epileptic activity.

The theory underlying EA is still controversial, although the action of EA has been widely discussed in literature. The discovery of endogenous opioid peptides, including enkephalin, β-endorphin, dynorphin and endormorphin, since 1970’s enhances the investigation of underlying mechanisms of EA, especially in the EA-induced analgesia. Three main receptor subtypes of the opioid receptors, including the μ-, δ- and κ-opioid receptors, in the spinal cord involve in the mechanisms of EA-induced analgesia. Endormorphin and dynorphin are respectively considered as the relatively pure μ- and κ-opioid receptor agonists [28,29], while enkephalin and β-endorphin are mixed μ- and δ- opioid receptor agonists (review [30,31]). Han and his colleagues have revealed that low frequency (2 Hz) EA increases met-enkephalin, but not dynorphin, in the spinal cord; while high frequency (100 HZ) EA increases the release of dynorphin rather than that of met-enkephalin [19]. The stimulation of EA between low and high frequency (e.g. 15 Hz) activates both enkephalins and dynorphins [19]. They further demonstrated that the analgesic effect induced by low-frequency EA stimulation is mediated by μ- and/or δ-opioid receptors; in contrast, high-frequency EA-induced analgesia is mediated by κ-opioid receptors [17,18]. These observations suggest that different endogenous opioid peptides would be released and act on distinct opioid receptors in the spinal cord under different stimulating conditions of EA. It remains unclear whether the endogenous opioid peptides and their receptors in the central nervous system (CNS) play a role in the epileptogenesis or they possess the anticonvulsant effect. Several studies indicate that opioid peptides inhibit the epileptic activity. For example, low doses of morphine or opioid peptides exhibit an anticonvulsant effect, which is blocked by low doses of naloxone [32]. Intracerebroventricular (ICV) administration of dynorphin suppresses the electroconvulsive shock- and kindled-induced seizure [33,34]. Temporal lobe epilepsy increases opioid receptors in the temporal neocortex in humans [35], which may mediate the anticonvulsant effects to limit the spread of electrical activity from other temporal lobe structures [34,36]. Furthermore, evidence that the prodynorphin knockout mice display a significantly reduced seizure threshold as assessed by administration of pentylenetetrazole suggests the anticonvulsant effect of endogenous opiates [37]. However, bulks of studies indicate that endogenous opioid peptides and their receptors contribute to the epileptogenesis. ICV injections of morphine and opioid peptides evoke pathological epileptiform EEGs [32,38-42]. β-endorphin could induce nonconvulsive limbic epileptiform activity in rats when the dose is devoid of analgesic and other behavioral signs [42]. Enkephalin and β-endorphin administered intracerebroventricularly or microinjected into discrete subcortical areas produce epileptic activities [32]. Repeated injection of small amounts of β-endorphin or met-enkephalin into the hippocampus or amygdala develops kindled generalized convulsions [39,40]. Dynorphin inhibits GABAergic neurons by activation of μ- and κ-opioid receptors, which results in the decrease of GABA release and facilitates seizure [38]. As for the opioid receptors, Carter et al. have demonstrated that opioid receptors are involved in the pathogenesis of a wide spectrum of seizure disorders [43]. Based on aforementioned evidence and our result of exacerbating pilocarpine-induced epileptiform activities by EA stimulation of Feng-Chi acupoints, we hypothesized that the EA-augmented epileptic activity is mediated by activation of opioid receptors in the CeA. Our results indicated that application of naloxone (a broad spectrum of opioid receptor antagonist), naloxonazine (a μ-receptor antagonist), naltrindole (a δ-receptor antagonist) or *nor*-binaltorphimine (a κ-receptor antagonist) significantly suppressed both the amplitude and the duration of EA-induced augmentation of epileptiform activity. The order of efficacy in suppressing EA-induced epileptiform EEGs was naltrindole > naloxone ≅ naloxonazine > *nor*-binaltorphimine. Activation of opioid receptors may mediate the EA-induced epileptic activities in the naïve rats; however, this needs to be further confirmed in the future study. Nevertheless, our current results favor the role of CeA opioid receptors in the epileptogenesis. Han and his colleagues demonstrate that the analgesic effects produced by low-frequency EA stimuli and high-frequency EA stimuli are mediated by different opioid receptors [17,30]. Our previous study also elicited that distinct opioid receptors in the NTS involve in different stimulation frequencies of EA-induced sleep enhancement [9,10], which is similar to the underlying mechanisms of EA-induced analgesia in the spinal cord as reported by Han and his colleagues. However, our current results indicate that 100 Hz EA stimulation of bilateral Feng-Chi acupoints activated μ-, δ- and κ-opioid receptors in the CeA to exacerbate pilocarpine-induced epilepsy. It is worthy to further investigate the effect of low-frequency (e.g., 10 Hz) EA stimulation of bilateral Feng-Chi acupoints on pilocarpine-induced epileptiform activity and the involvement of opioid receptors in the CeA. Strategy by employing pharmacological blockade to elucidate the involvement of particular opioid receptors in 100 Hz EA-induced augmentation of epileptiform activity is appropriate. However, it would be of interest to mimic the EA-induced epileptiform EEGs by microinjection of opioid-receptor agonists, e.g. β-endorphin, encephalin and dynorphin, into the CeA in future.

## Conclusion

In summary, our current results indicated that high-frequency (100 Hz) EA stimulation of bilateral Feng-Chi acupoints exacerbated pilocarpine-induced focal epilepsy. The EA-induced exacerbation of focal epilepsy was blocked by administration of μ-, δ-, or κ-opioid receptor antagonist into the CeA, demonstrating the involvement of CeA opioid receptors. Our current results suggest that 100 Hz EA stimulation of bilateral Feng-Chi acupoints did not exhibit effect to against epilepsy, whereas it further deteriorated the focal epilepsy.

## Competing interests

The authors declare that they have no competing interests.

## Authors’ contributions

PLY, CYL and YFT carried out the experiments. PLY and CYL analyzed sleep data. PLY, CHC, CTL and FCC designed the experimental protocols. PLY and FCC prepared the manuscript. All authors read and approved the final manuscript.

## Pre-publication history

The pre-publication history for this paper can be accessed here:

http://www.biomedcentral.com/1472-6882/13/290/prepub
